# Engineered Antibodies to Improve Efficacy against Neurodegenerative Disorders

**DOI:** 10.3390/ijms25126683

**Published:** 2024-06-18

**Authors:** Sarfaraz K. Niazi, Zamara Mariam, Matthias Magoola

**Affiliations:** 1 College of Pharmacy, University of Illinois, Chicago, IL 60612, USA; 2 Centre for Health and Life Sciences, Coventry University, Coventry City CV1 5FB, UK; mariamz@coventry.ac.uk; 3 DEI Biopharma, Kampala P.O. Box 35854, Uganda; dei@deiinternational.com

**Keywords:** neurodegenerative disorders, transcytosis, transferrin, Alzheimer’s disease, minibodies, Fab fragments, nanobodies, amyloid-β, α-synuclein

## Abstract

Antibodies that can selectively remove rogue proteins in the brain are an obvious choice to treat neurodegenerative disorders (NDs), but after decades of efforts, only two antibodies to treat Alzheimer’s disease are approved, dozens are in the testing phase, and one was withdrawn, and the other halted, likely due to efficacy issues. However, these outcomes should have been evident since these antibodies cannot enter the brain sufficiently due to the blood–brain barrier (BBB) protectant. However, all products can be rejuvenated by binding them with transferrin, preferably as smaller fragments. This model can be tested quickly and at a low cost and should be applied to bapineuzumab, solanezumab, crenezumab, gantenerumab, aducanumab, lecanemab, donanemab, cinpanemab, and gantenerumab, and their fragments. This paper demonstrates that conjugating with transferrin does not alter the binding to brain proteins such as amyloid-β (Aβ) and α-synuclein. We also present a selection of conjugate designs that will allow cleavage upon entering the brain to prevent their exocytosis while keeping the fragments connected to enable optimal binding to proteins. The identified products can be readily tested and returned to patients with the lowest regulatory cost and delays. These engineered antibodies can be manufactured by recombinant engineering, preferably by mRNA technology, as a more affordable solution to meet the dire need to treat neurodegenerative disorders effectively.

## 1. Introduction

Neurodegenerative diseases (NDs) are complex disorders with multifactorial pathology that result in progressive damage to neuronal cells and loss of neuronal connectivity, ultimately leading to impaired mobility and cognition. Protein aggregation due to misfolding and oligomerization gives rise to extracellular or intracellular inclusions, a common hallmark for many NDs. Further spreading, such as those of amyloid aggregates in the nervous system, are like prion-based infections; hence, a prion-like mechanism is often considered a significant element in the etiology of NDs [1].

In NDs, traditionally considered distinct from autoimmune disorders, recent research has begun to highlight significant interactions between neurodegeneration and the immune system, particularly involving T-cell responses. This emerging understanding complicates the conventional classification of these disorders. NDs such as Alzheimer’s disease (AD) and Parkinson’s disease (PD) are characterized by the progressive loss of neural function and structure, leading to severe cognitive and motor impairments. Traditionally, these disorders were not considered to involve autoimmune mechanisms. However, evidence now indicates that immune responses, including those mediated by T cells, are indeed involved in the pathology of these diseases. For instance, in AD, T cells have been found near amyloid plaques, suggesting an immune reaction to the disease’s progression [2].

Similarly, in PD, immune cells accumulate in response to neuronal death or abnormal protein aggregates [3]. Chronic inflammation is common in these diseases, often driven by an ongoing immune response. This inflammation can exacerbate neuronal damage, contributing to the disease’s progression. The role of T cells in this context is significant but appears to be more about responding to disease pathology rather than initiating it. This distinction is crucial as it differentiates reactive immunity from the self-targeting characteristic of classical autoimmune diseases where the immune system mistakenly attacks healthy cells. Multiple sclerosis (MS) stands out as a clear example of a neurodegenerative disease that is also classified as an autoimmune disorder. In MS, the immune system directly attacks the myelin sheath of nerve fibers, a clear autoimmune response. This direct immune involvement in MS contrasts with the more secondary role that immune responses play in other neurodegenerative diseases. Understanding the interplay between neurodegeneration and immune responses, especially T-cell involvement, is essential for developing effective treatments. The goal is to modulate these immune responses to prevent or reduce damage without exacerbating the underlying disease processes.

This nuanced view of the immune system’s role in neurodegenerative diseases fosters new research directions to delineate the specific contributions of immune components to these disorders. Further research will likely continue to blur the lines between neurodegenerative and autoimmune classifications, potentially leading to novel therapeutic approaches that address both the degenerative and immune aspects of these complex diseases [4].

According to the Alzheimer’s Association’s “2023 Alzheimer’s Disease Facts and Figures” report, an estimated 6.7 million Americans aged 65 and older are living with Alzheimer’s dementia today. This number could grow to 13.8 million by 2060, barring the development of medical breakthroughs to prevent, slow or cure AD [5].

In the past few decades, many of the genetic and biochemical causes underlying NDs associated with protein aggregation were uncovered, leading to the distinction between rarer familial forms, where disease-causing mutations are genetically inherited, and the more common sporadic forms, where genetic and environmental risk factors drive the pathogenesis [6]. In both cases, the affected proteins are found enriched in pathological aggregates, highlighting their importance in the manifestation of the disease. Details of various pathways leading to NDs are well presented in a KEGG diagram [7].

In AD, two different types of deposits are observed. The aberrant cleavage products of the transmembrane protein Aβ precursor protein (APP) form extracellular plaque deposits in the temporal and parietal brain regions. In contrast, the protein tau (MAPT) accumulates intracellularly in neurofibrillary tangles [8].

In Parkinson’s disease (PD), the primarily affected brain area is the substantia nigra (SN), where α-synuclein (α-syn; SNCA) aggregates are found to accumulate in dopaminergic neurons [9].

In ALS, cellular aggregates of superoxide dismutase 1 (SOD1), RNA-binding protein FUS (Fused in Sarcoma), and TAR DNA-binding protein 43 (TDP-43) have been identified in motor neurons of the primary motor cortex, brainstem, and spinal cord [10]. It was in 2011 when the non-coding repeat expansion in the C9orf72 gene was discovered to be the most frequent cause of frontotemporal dementia (FTD) and amyotrophic lateral sclerosis (ALS) [11,12]; this gene and its derived protein, C9orf72, were utterly unknown. The mutation appeared to produce both haplo-insufficiency and gain-of-function effects in the form of aggregating expanded RNAs and dipeptide repeat proteins (DPRs)—elements that should be taken into consideration to refine future therapeutic strategies, compensating for the decrease in C9orf72 or at least preventing a further reduction [11,12].

However, despite the accumulated knowledge and the many clinical, no therapeutic strategy has proven successful to cure any of the NDs. This led many scientists even to question whether protein aggregation is central to ND etiology or a manifestation of other underlying causes [13,14]. However, despite failures, the developers keep repeating the testing of similar drugs, even when the failures are apparent, perhaps due to the lack of other options and the need to have an active portfolio for one of the most lucrative markets for new drugs.

## 2. Current Status

The BBB is formed, in part, by endothelial cell tight and adherent junction proteins, which severely restricts the transport of most macromolecules circulating within the blood compartment (2–6). This limited exchange represents a key challenge for delivering peripherally administered protein therapeutics into the CNS. Antibody bioavailability in the brain is low, often cited to be around 0.1% of circulating serum concentrations [15]. Multiple groups have observed that CNS exposure for circulating biologics is limited to 0.1 to 0.4% of corresponding serum concentrations [16,17,18], although some estimates for transporting immunoglobulins into neural tissue are far lower [19]. Consequently, the maximal brain concentration for peripherally dosed large molecules is often insufficient to achieve the target engagement required for a therapeutic response [20,21]. Administration methods such as intracranial, intrathecal, or intraventricular injections and chemical BBB disruption may facilitate higher brain concentrations for some drugs. Still, these approaches are invasive and incompatible with repeat dosing regimens [22,23,24]. Although human genetics combined with rodent disease models has improved our ability to select promising biological targets for many drug candidates, insufficient brain exposure remains a likely cause for the failure of many antibodies and other larger-size molecule drugs directed toward CNS diseases over the past decade [25].

Antibodies designed to reduce protein deposits in the brain, specifically amyloid-beta (Aβ) plaques, are tested by administering exogenous antibodies (passive immunization) that can clear Aβ from the brain and thus halt the neurodegenerative progression, assuming that the activation of microglial phagocytosis via Fc-effector functions will prove out to be the primary mechanism of treatment [26].

In 2021, the FDA conditionally approved the first such therapy, Aducanumab, Aduhelm^®^) [27]. This human IgG1 monoclonal antibody targets amyloid-beta plaques. However, the decision was controversial [28]. It was made under an accelerated approval process [29,30] where the cognitive decline was only slowed in one of two studies at the highest dose [31]; soon after the approval, it was discontinued, effective in 2024, without reasons and with the claim that it was not due to issues with safety or efficacy [32]. Aducanumab’s approval was significant as it was the first disease-modifying therapy (DMT) for AD approved in two decades [33].

A second therapeutic antibody, lecanemab (Leqembi^®^), was recently granted the same type of approval (January 2023) by the FDA. Lecanemab is the humanized version of murine mAb158 with high selectivity for soluble protofibrils [34]. The approval was based on phase 2 clinical data that lecanemab reduced Aβ-plaque load [35]. However, results in the phase 3 study slowed overall cognitive decline by 27%. They had positive outcomes on biomarkers and secondary endpoints, further providing evidence of aggregated Aβ as a viable target for treatment in AD [36,37]. The FDA approved lecanemab in January 2023 but halted further progress on lecanemab after the withdrawal of aducanumab.

Donanemab is also under development to treat AD [38], and cinpanemab is under clinical testing for PD [39]; its development is halted by the FDA [40] likely due to a lack of proof of efficacy [41]. Two other anti-Aβ-antibodies are evaluated in phase 3 clinical trials: donanemab and gantenerumab. While more selective for aggregated Aβ than monomers aducanumab and lecanemab, their binding profiles differ slightly within the spectrum from soluble to fibrillary Aβ [42].

Cinpanemab is one of several α-synuclein antibodies being investigated for PD [43] It is intended to treat ALS and was recalled due to lack of efficacy [44,45].

For AD treatment, there are also clinical trials MEDI1814, ADEL-Y01, TB006, PF-04360365, and LY2062430, among others.

Others for PD include ABBV-0805, MEDI1341, LU AF82422, and prasinezumab (https://www.alzforum.org/therapeutics/cinpanemab, accessed on 7 April 2024).

Clinicaltrials.gov lists around 250 interventional studies that treat NDs involving antibodies [46]. However, it seems highly unlikely that these trials will fulfill the gaps created by the recent setbacks in the use of full-size antibodies to treat neurodegenerative disorders since these antibodies cannot cross the blood–brain barrier (BBB) without any structural modifications to the antibody or using adjuvant means to improve their entry across the BBB.

In the past, several antibody candidates have failed to meet clinical endpoints^104^. Bapineuzumab (the humanized version of the murine 3D6) was the first anti-Aβ antibody that entered clinical trials. It binds to the N-terminal end (amino acid 1–5) and thus binds all forms of Aβ [47]. Despite showing reductions in Aβ plaques, bapineuzumab studies were terminated because they lacked treatment effects in two phase 3 trials. Side effects related to vascular Aβ were also raised as a concern [48].

The failure of bapineuzumab and other first-generation anti-Aβ antibodies has been attributed to several reasons. Firstly, many patients were not Aβ-plaque positive in the bapineuzumab study owing to a lack of screening and could have suffered from a different type of dementia [49]. Secondly, patients might have been too advanced in their pathology, and thus, it was too late to slow cognitive decline. Later studies of aducanumab and lecanemab have included patients with generally higher cognition scores [36]. Thirdly, early Aβ antibodies targeted monomeric anti-Aβ antibodies; it is possible that targeting Aβ aggregates is more beneficial. Lastly, antibodies might not have reached the target within the brain to a high enough concentration because of too low dose or overall low brain delivery. As a result, gantenerumab doses have been increased in later studies [50], and the highest and most frequently dosed antibodies have so far proved to be the most efficient in halting cognitive decline, suggesting that high brain concentrations are needed to improve symptoms [51].

However, high doses also increase the risk of side effects. The main side effect of anti-Aβ antibodies is “amyloid-related imaging abnormalities” or ARIA, identified by MRI, classified into two subtypes, ARIA-E (edema) and ARIA-H (hemorrhage) [52]. The condition can be asymptomatic; if symptoms occur, they are often mild and temporary. However, some patients who experienced more severe symptoms have been withdrawn from the clinical trials. There is a higher risk of developing this side effect for carriers of APOE-ε4 [53] and having two copies of this gene virtually guarantees the development of Alzheimer’s at an earlier age [54,55].

Enhancing the brain delivery of therapeutic antibodies to treat NDs could increase efficacy and lower the risk of side effects like ARIA for future immunotherapies [56]. However, there has been variation in the ability of the monoclonal anti-Aβ antibodies studied in large-scale AD trials to lower insoluble brain Aβ levels (PET-detectable). However, PET is not able to distinguish between parenchymal and vascular amyloid. Therefore, while data showing how much anti-Aβ monoclonal antibodies have decreased PET-detectable Aβ in clinical trials probably primarily reflect these antibodies’ effects on neuritic plaques, the data are not exclusive to neuritic plaques as there may also have been some clearance of diffuse plaques and/or CAA-associated Aβ.

A recent meta-analysis reports thirty-three randomized controlled trials with 21,087 patients involving eight mAbs. Despite immunotherapies significantly increasing the risks of adverse events and ARIA, the data suggest that mAbs can effectively improve the cognitive function of patients with mild and moderate AD. According to the NMA, aducanumab was the most likely to achieve significant improvements in different cognitive and clinical assessments (statistically improved MMSE and CDR-SB), followed by donanemab (statistically improved ADAS-Cog and PET-SUVr) and lecanemab (statistically improved ADCS-ADL) [57].

Table 1 lists monoclonal anti-Aβ antibodies whose structural details, although incomplete, are available; KEGG provides the whole sequences and, in one case, only heavy chain and none for bapineuzumab. PDB only reports complex Fabs with Aβ in some cases, which helps establish the binding efficacy, as shown below when connected to transferring protein.

### 2.1. Amyloid Hypothesis

Protein misfolding and aggregation of disease-associated proteins are facilitated by mutations and post-translation modifications (e.g., phosphorylation and protein cleavage) that avert the formation of the native protein structure.

No gene variant is a more significant AD risk factor than APOE4. However, exactly how the gene spurs brain damage has been a mystery [70]. One study [71] has now linked APOE4 with faulty cholesterol processing in the brain, leading to defects in the insulating sheaths that surround nerve fibers and facilitate their electrical activity. Inheriting a single copy of APOE4 raises the risk of developing Alzheimer’s disease (AD) around 3-fold; having two copies boosts the chances 8- to 12-fold.

Different genes are typically found mutated in the familial forms, as they initially affect other brain regions and cell types. For example, Huntington’s disease (HD) and spinocerebellar ataxia type 1 (SCA1) are linked to the expansion of the CAG (Cytosine, adenine, guanine) repeat of the huntingtin (HTT) and ataxin 1 (ATXN1) genes, respectively, results in proteins with an unusually long polyglutamine (polyQ) tract that is highly prone to aggregation and causing intracellular deposits in striatal neurons [72,73].

Misfolding can also occur sporadically without a clear explanation. Aggregation is first typically initiated by a seed or an oligomer, in which sequence-specific elements of the misfolded protein interact to adopt a non-native conformation, which can then convert other proteins into the toxic form. In many cases, the oligomerization of misfolded proteins leads to the formation of amyloid fibrils with a distinctive β-sheet structure that arises when soluble oligomers assemble into small protofibrils [74]. When more proteins are converted into non-native forms, these protofibrils become longer fibrils that can then form more extensive cellular inclusions visible by light microscopy. Recently, it has been proposed that oligomerization may be favored by liquid–liquid phase separation of aggregation-prone proteins [75].

Moreover, it is evident that there are different polymorphs for most amyloid fibrils in vitro and in vivo (polymorph is a term used to indicate the capacity of a polypeptide to generate fibrils with different structures) [76,77]. The protein fibrils formed would be expected to be removed by the autoimmune responses. Still, the less efficient production of antibodies in the brain and lack of entry of systemic antibodies requires promoting systemic entry across the blood–brain barrier (BBB) [78,79].

AD’s two main neuropathological characteristics are tau protein-containing neurofibrillary tangles and Aβ plaques. The plaques can be classified as diffuse plaques with filamentous Aβ and without dense cores or as neuritic plaques (also called senile plaques (SP) with tau-paired helical filament including neurites and dense cores of fibrillar Aβ [80]. Neurites are often absent from diffuse plaques but can appear in late-stage AD [81].

The amyloid hypothesis proposed that increased fibrillar Aβ deposition was the cause of AD-type disease and was published by Hardy and colleagues in the early 1990s [82]. However, the findings of increased SP densities in some subjects with little or no cognitive impairment, weak correlations between Aβ-targeting approaches failing in large-scale AD clinical trials, and insoluble Aβ levels and PET-detectable Aβ with measures of cognitive impairment all cast doubt on the hypothesis [83].

Since the concept was published, decreasing brain Aβ has been the focus of efforts to halt AD progression. Enzymatic breakdown and brain efflux are two ways that Aβ might be eliminated from the brain. Aβ is eliminated from the brain by the blood–brain barrier (BBB) [84], the blood–cerebrospinal fluid (CSF) barrier [85], glymphatic (paravascular) drainage [86], and perivascular drainage [87]. It is broken down by the endosomal-lysosomal system [88], the ubiquitin–proteasome system [89], and autophagy [90].

Recent findings indicate that the main factor underlying the development and progression of AD is tau, not Aβ and to delay the course of AD, the downstream neuropathological processes like tau phosphorylation and aggregation, as well as (maybe) oxidative stress and inflammation [91], must be controlled. Aβ oligomers may be the first to cause AD pathology, according to a later revision of this idea [92,93]. There have also been suggestions that Aβ might not be the best or most appropriate treatment target to halt the course of AD [94].

Another observation is that BBB degradation occurs early in AD [95], likely increasing peripheral blood IgG penetration into the brain [96]. This is why the treatment of NDs is more effective in the early stages [97], it is suggested.

A significant focus of this paper is to suggest methods to enhance the delivery of antibodies by letting them piggyback with proteins that bind to endothelial membranes and create transcytosis that will allow the entry of antibodies if conjugated with these proteins.

### 2.2. Brain Immunity

An obvious choice to treat NDs is to neutralize the antigens with antibodies, a typical response in the systemic circulation but not available in the brain. Although the BBB restricts the entry of peripheral immune cells into the brain under normal conditions, T and B cells can cross into the CNS under certain circumstances, such as inflammation or BBB breakdown. Once in the brain, these cells can contribute to immune surveillance and the pathogenesis of neuroinflammatory diseases.

A major immunity pathway in the brain depends on microglia, which provide macrophages in the brain [98]. Microglial Fc receptors (FcR) attach to antibodies via their Fc (“fragment crystallizable”) sections when the antibodies bind via their Fab (“fragment antigen-binding”) regions to fibrillar Aβ [99]. Microglia may be protective by promoting phagocytosis and removal of Aβ deposits, but also become dysfunctional as the disease progresses, producing neurotoxins, ceasing to clear Aβ deposits, and producing cytokines that further promote Aβ deposition [100,101]. It has been shown that in Alzheimer’s disease, Aβ directly activates microglia and other monocytes to produce neurotoxins [102]. Preclinical and clinical studies have shown that cellular (microglia/macrophages, leukocytes, astrocytes, mast cells, etc.), and molecular neuroimmune responses contribute to secondary brain injury after intracerebral hemorrhage [103,104].

Fibrillar Aβ has the ability to activate both the traditional and alternative complement pathways, hence facilitating microglia absorption [105]. Binding of C1q to the antibodies can promote microglial absorption of antibody–Aβ complexes when anti-Aβ antibody levels are not ideal for boosting microglial phagocytosis of these complexes [106]. Complement activation has been described as having two opposing effects: although complete complement activation produces the neurotoxic membrane assault complex C5b-9, early complement activation proteins facilitate the clearance of Aβ [107].

### 2.3. Natural Antibodies

AD patients and others who are not cognitively impaired may already have peripheral blood and the CSF filled with antibodies that could bind to Aβ. Natural antibodies to Aβ (Nabs-Aβ) are used to describe these antibodies [108]. Natural antibodies can be produced without antigenic stimulation, meaning they may not be fully antigen-specific [109].

Nabs-Aβ is a component of IVIG products made from plasma immunoglobulins from many (often more than 10,000) healthy donors [110]. IVIG or its pure anti-Aβ antibodies have been shown to have Aβ-related effects, such as breaking down Aβ fibrils and encouraging Aβ phagocytosis [111], preventing Aβ oligomer formation [112], and shielding SH-SY5Y neuroblastoma cells from the harmful effects of Aβ oligomers [113]. Apart from Nabs-Aβ, IVIG products also include antibodies against phosphorylated tau (p-tau-199 and p-tau-202) and non-phosphorylated tau (recombinant human tau peptide, Tau-441, 2N4 R) [114].

The creation of transgenic mice that express the human APP, presenilin 1, and presenilin 2 gene mutations linked to early onset AD [115] prompted research on the effects of systemic administration of monoclonal anti-Aβ antibodies for passive immunization and Aβ vaccination for active immunization.

Antibodies further assist cerebral Aβ removal through “peripheral sink” activity [116]. Clearing the Aβ from the CSF continuously (the “CSF-sink” therapeutic strategy) when soluble peptides are in constant equilibrium between the ISF and the CSF, altering the levels of Aβ oligomers in the CSF alters the levels of such proteins in the brain parenchyma, producing a steady clearance of Aβ in the ISF [117].

In addition to stimulating microglial phagocytosis, anti-Aβ antibodies have been demonstrated to lower brain Aβ through several pathways. Microglia are unnecessary for immunotherapeutic clearance of surface proteins [118]; the uptake of anti-Aβ antibodies via microglial FcR does not account for the majority of antibody-mediated clearance of surface proteins [119].

Anti-Aβ antibodies have also been shown to inhibit Aβ aggregation [120]; other non-microglial mechanisms of antibody-mediated Aβ clearance include dissolving Aβ aggregates [121] and facilitating the efflux of antibody–Aβ aggregates from the brain via the BBB, following their binding to BBB receptors such as LRP1 and the neonatal FcR [122].

Monoclonal antibodies bind to various regions of Aβ. These include its central domain, its C-terminal residues (whose binding by antibodies in peripheral blood should result in Aβ being sequestered in peripheral blood [123]), its N-terminal amino acids (which affect Aβ’s ability to aggregate [124] and are accessible for antibody binding to fibrillar β [125], and conformation-specific epitopes like pyroglutamate-bound Aβ, which is present on SPs [126]. The Aβ peptide exists in several forms, including full-length Aβ_1–42_ and Aβ_1–40_—and the N-truncated species, pyroglutamate Aβ_3–42_ and Aβ_4–42_, which appear to play a significant role in neurodegeneration. A β-hairpin structure in the N-terminal region of Aβ is also identified [127].

An interesting observation is that aged mice show poorer brain delivery of the bispecific antibody, mAb3D6-scFv8D3, compared with younger mice. Age was also related to increased blood cell binding of the bispecific antibody, and a lower dose resulted in higher relative delivery to the brain parenchyma [128]. Whether it applies to humans remains to be discovered.

## 3. Engineered Antibodies

Due to IgG’s enormous molecular weight (about 150 kDa), peripheral blood IgG can virtually never enter the brain in normal mice; barely 0.1% of systemically injected IgG can pass across the blood–brain barrier [15]. However, smaller antibody fusions are eliminated faster from blood and cleared from the brain earlier than the large antibody, making more significant antibodies a better choice for brain PET analysis [129] and efficacy.

Several antibody fragments have been approved by the FDA for therapeutic use, such as Fabrazyme (agalsidase beta), Lucentis (ranibizumab), Cimzia (certolizumab pegol); Adecatumumab, Abciximab, Idarucizumab, and Digoxin immune Fab.

**Figure 1:**
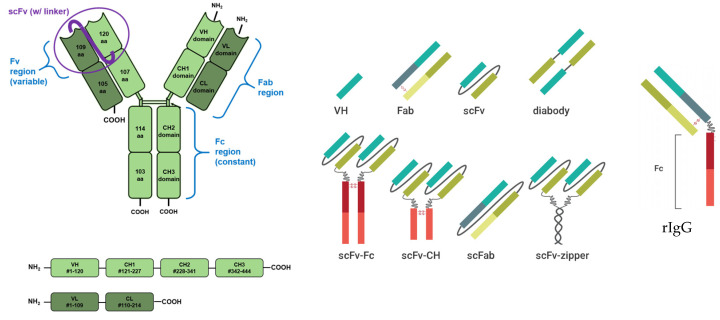
Domains and regions of a full and shortened IgG.

Figure 1 shows a variety of smaller antibodies that retain the function of the whole antibody.

### 3.1. scFv-CH (Single-Chain Variable Fragment)

This is the smallest functional unit of an antibody that can still bind to its target antigen. It consists of the variable regions of the heavy (VH) and light (VL) immunoglobulin chains, linked together by a short peptide linker. This structure enables the scFv to maintain the specificity and affinity of the original antibody. Fc is the tail region of an antibody that interacts with cell receptors and the complement system, which are part of the immune response.

### 3.2. Single-Chain Variable Fragment (scFv)

scFv, a fusion protein, is only half the size of the Fab fragment, approximately 25 to 30 kDa [130], yet retains the original specificity of the parent immunoglobulin. The VH and VL can be connected to a short linker peptide of 10 to about 25 amino acid chains [131]. Unlike monoclonal antibodies, these antibodies are generally produced in bacteria cell cultures such as *E. coli*. An example of scFv is HexaRmAb158, a single-chain multivalent fragment variable that targets and binds more efficiently to soluble aggregates and small oligomers of Aβ [132]. scFv-CH (single-chain variable fragment and constant heavy chain domain).

An antibody’s CH (constant heavy domain) part encompasses one or more domains of the heavy chain’s constant region, excluding the Fc region used in scFv-Fc configurations. Depending on the desired properties and the specific application, the CH domain can include one or several domains, such as CH1, CH2, and CH3. Each domain can contribute differently to the fusion protein’s stability, solubility, and dimerization properties.

### 3.3. scFV-CH3 (Minibodies)

Minibodies are scFvs linked to the CH3 domain of an antibody’s Fc region, demonstrating pronounced cytotoxic effects and strong antigen binding compared to scFv-based antibody fragment–drug conjugates. Including the CH3 domain allows the minibodies to dimerize, mimicking the bivalent (two-antigen-binding sites) nature of full antibodies, which can improve binding strength and specificity. This indicates their potential for enhancing therapeutic efficacy in antibody–drug conjugate applications by improving pharmacokinetics and reducing side effects [133]. Minibodies can be produced relatively quickly in various expression systems, such as yeast or mammalian cells, which can be more cost-effective and scalable than full antibody production.

### 3.4. Diabody

A diabody is two scFvs connected with linker peptides that are too short for the two variable regions to fold together (about five amino acids), forcing the scFvs to dimerize; they have dissociation constants up to 40-fold lower than corresponding scFvs, yielding a much higher affinity to their target.

### 3.5. sdAb (Single Domain Antibody)

A single-domain antibody (sdAb), a nanobody, is not derived from human IgG. With a molecular weight of only 12–15 kDa, these are even smaller than Fab fragments (~50 kDa) [134] having a peptide chain of about 110 amino acids long and only one variable domain (VH) of a heavy-chain antibody. Their smaller size allows use for imaging, diagnostics, and therapeutics, as they have better tissue penetration and can reach epitopes that are inaccessible to more significant antibodies. An alternative approach is to split the dimeric variable domains from common immunoglobulin G (IgG) from humans or mice into monomers. Nanobodies derived from light chains have also been shown to bind specifically to target epitopes [135]. Caplacizumab is the first approved nanobody drug, marking a significant advancement in nanobody-based therapy, showcasing its potential for disease treatment [136]. One groundbreaking study by researchers from Johns Hopkins University demonstrated a nanobody’s ability to penetrate brain cells and untangle misshapen proteins that lead to PD, potentially halting the progression of the disease. This nanobody, PFFNB2, could bind to alpha-synuclein clumps, preventing their harmful effects on the brain [137].

### 3.6. F(ab)2 Fragments

The F(ab’)2 fragment is a specific antibody obtained by enzymatic cleavage. An antibody, or immunoglobulin, is a protein the immune system uses to identify and neutralize foreign objects like bacteria and viruses. The typical antibody structure can be divided into several parts, with the F(ab’)2 fragment comprising a significant portion. The F(ab’)2 fragment includes two antigen-binding Fab fragments connected by a hinge region. Each Fab fragment consists of one constant and variable domain from the heavy and light chains.

### 3.7. F(ab) Fragment

The F(ab’) fragment is a specific type of antibody fragment that includes the antigen-binding portion of the antibody. Unlike the F(ab’)2 fragment, which consists of two linked Fab units, the F(ab’) fragment is a single Fab unit with an additional tail at the hinge region. The F(ab’) fragment consists of one arm of the antibody, which includes a variable region (V) and a constant region (C) from both the light chain and part of the heavy chain. The ‘tail’ included in the F(ab’) fragment extends from the hinge region where the antibody would normally be cleaved to separate it from the Fc portion. Fab fragment size typically ranges from approximately 50 to 60 kDa [138]. One example of an antibody fragment under development for treating neurodegenerative disorders is “ALZ-801”, also known as “Azeliragon”. It is an oral, small-molecule inhibitor of beta-amyloid aggregation that is currently in clinical development for Alzheimer’s disease. It is derived from a monoclonal antibody called “3D6”, which targets beta-amyloid.

### 3.8. Reduced IgG (rIgG)

The rIgG refers to reduced IgG (75,000 Daltons) or half-IgG; the most significant advantage of these antibodies is that they can be readily constructed without the need to identify the variable zones. These half-molecules consisting of one heavy and one light chain appear to be a rare antibody [139] antigenically deficient in the Fc fragment. Being only half, it is not interactive. Half-IgG are frequently prepared to target the exposing hinge-region sulfhydryl groups that can be targeted for conjugation, either antibody immobilization or enzyme labeling [140].

### 3.9. Bispecific Antibodies (BsAbs)

BsAbs have two distinct binding domains that can bind simultaneously to two antigens or two epitopes (an antigen part) of the same antigen; these are next-generation antibodies that can induce multiple physiological or pathological responses; these treatments may act like a “cocktail”. The current focus of BsAbs is to treat cancer, but it is expanding to include chronic inflammatory, autoimmune, neurodegenerative diseases; vascular, ocular, and hematologic disorders, and infections. Since 2014, the FDA has approved nine BsAb marketing applications to treat cancer and hematologic and ocular diseases, and the FDA anticipates many BsAbs coming to patients [141], as there are over 100 BsAbs in clinical development, most in the early stages [142].

### 3.10. Multispecific Antibodies

Multispecific antibodies, which can target multiple antigens simultaneously, are also moving forward. There is a dizzying array of potential formats for multispecific antibodies, which may lead to treatments for diseases with no or few therapies. FDA will continue to help move the needle in this therapeutic area. Examples of FDA-approved bispecific antibodies include Blincyto blinatumomab; Hemlibra, Rybrevant; Kimmtrak, Vabysmo; Tecvayli; Lunsumio; Epkinly; Columvi; and Kimmtrak, which is technically a bispecific molecule, not a bispecific antibody.

## 4. Administration and Disposition Profile

The BBB controls brain homeostasis, allowing the passage of molecules necessary for brain cell function. Some essential molecules transported across the BBB are glucose, hormones, vitamins, insulin, leptin, and iron [143]. While the smaller antibody types, as described above, can significantly enhance their entry across the BBB, the whole antibodies may require invasive techniques, including intra-cerebral injection, convection-enhanced delivery, and intra-cerebroventricular infusion [144] to achieve therapeutic levels in the brain. Additionally, the BBB can be disrupted using bradykinin analogs, ultrasonography, and osmotic pressure [145]. Adding microbubbles makes these techniques more effective [146,147]. Pharmacological techniques involve encapsulating medications into liposomes or chemically modifying pharmaceuticals to lipophilic molecules [148]. Opsonization and drug delivery by nanoparticles across the blood–brain barrier, in which the drug is adsorbed onto the particles passively [149]. Intranasal delivery routes can bypass the BBB, offering a direct path to the CNS [150]. Also investigated are vaccines considering Aβ aggregates as antigens [151], Aβ aggregation inhibition [152], β-secretase inhibition [153], γ-secretase modulation [154], *γ*-secretase inhibition [155], intravenous immunoglobulin (IVIG) administration [156], and most widely introducing monoclonal anti-Aβ antibodies [157]. However, none of these strategies have met primary end goals in large-scale clinical trials.

Another strategy involves administering an antibody-like lecanemab, which targets soluble Aβ (protofibrils are large, oligomeric species) [158]. This antibody would be paired with an intervention to boost peripheral sink efflux of Aβ from the brain. Increasing peripheral blood levels of sLRP1, administering a monoclonal antibody that binds to the C-terminal residues of peripheral blood Aβ to sequester it, and plasmapheresis with albumin replacement appears plausible, as shown in the AMBAR study [159].

The systemic clearance of antibodies typically involves pathways like pinocytosis and interactions with the FcRn receptor, which recycles antibodies and prolongs their half-life; the BBB restricts the entry and exit of substances, including antibodies. Clearance mechanisms in the brain include exocytosis across the BBB and potentially the glymphatic system, which facilitates the clearance of waste from the central nervous system during sleep. This distinction reflects the brain’s specialized protection and maintenance requirements [160]. Antibody systemic clearance highly depends on size, as shown in Table 2 [161].

The shorter systemic half-life for smaller molecules is primarily due to renal clearance through glomerular filtration. Their small size (usually less than 60 kDa) allows them to be filtered through the kidney glomeruli, unlike larger molecules retained in the bloodstream.

The clearance of these small antibody fragments from the brain involves mechanisms somewhat different from those in the bloodstream. Within the brain, the movement of molecules through the extracellular space is influenced by interstitial fluid flow, which can lead to the clearance of molecules via bulk flow. This fluid eventually drains into the cerebrospinal fluid (CSF) and is cleared from the brain. Due to their size, small antibody fragments may more readily diffuse through the interstitial space and be cleared through this pathway compared to larger molecules. Recent studies have identified lymphatic vessels that line the dural sinuses of the brain. These vessels drain interstitial fluid and macromolecules from the CSF and brain interstitium to the deep cervical lymph nodes. This lymphatic system can contribute to clearing small antibody fragments from the brain. The brain endothelium expresses various efflux transporters, such as P-glycoprotein (P-gp) and other ATP-binding cassette (ABC) transporter family members, which can actively transport a wide range of substances from the brain. If small antibody fragments interact with these transporters, they could be subject to active efflux, similar to many small-molecule drugs.

Some small molecules are also actively removed from the endothelial cells by efflux pumps, e.g., p-glycoprotein (P-pg). Antibodies may also be effluxed from the brain via the FcRn receptor [162], but this is debated, e.g., FcRn-deficient mice do not have significantly higher brain levels of IgG [163].

Like other proteins, antibody fragments can be metabolically degraded by enzymes within the brain. The resulting peptides and amino acids may then be reused by brain cells or removed by other clearance mechanisms.

### 4.1. Transcytosis

Transcytosis of antibodies and their fragments can occur through several mechanisms, including receptor-mediated transcytosis (RMT), adsorptive-mediated transcytosis, and cell-mediated transcytosis [164,165]. One of the most exploited receptors for RMT is the transferrin receptor (TfR), which naturally facilitates iron transport into the brain. Antibodies designed to target the TfR can hitch a ride across the BBB through this mechanism. Another example is the insulin receptor, which has also been targeted for RMT to deliver antibodies into the brain.

Another target is the insulin receptor (IR), which, like the TfR, can mediate the transport of antibodies across the BBB, offering a pathway for therapeutic intervention [166]. So, theoretically, a conjugate of insulin and an antibody should enhance the entry of antibodies across the BBB, though no such studies have been reported.

The low-density lipoprotein receptor-related protein-1 (LRP1) also serves as a conduit for the delivery of certain therapeutics into the brain, capitalizing on its role in transporting various molecules, including lipoproteins and amyloid-beta precursors [84].

The other two proteins, GLUT1 and P-glycoprotein (P-gp), have not been well studied or found effective [167].

Caveolae are specialized invaginations of the plasma membrane that play a role in various cellular processes, including endocytosis, transcytosis, and signal transduction. In the context of the BBB, caveolae-mediated transcytosis represents a potential mechanism for the transport of specific molecules, including antibodies, across the BBB [168].

However, the exact mechanism that can take them into the brain also enters the bloodstream. Although less likely for larger molecules, any small and sufficiently lipophilic molecules might also diffuse passively across cellular membranes, moving from areas of higher concentration (in the brain interstitial fluid) to lower concentration (in the blood).

### 4.2. Transferrin

**Figure 2:**
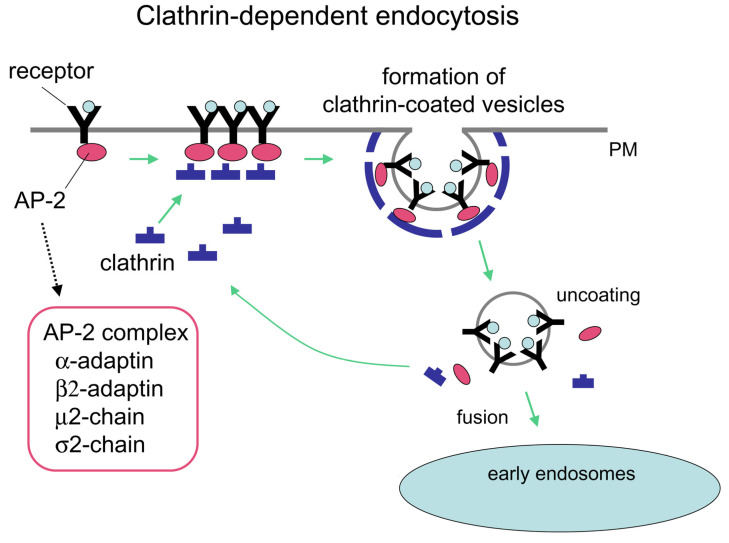
Clathrin-based endocytosis. (By Grant, B. D. and Sato, M—http://www.wormbook.org/chapters/www_intracellulartrafficking/intracellulartrafficking.html, accessed on 7 March 2024. (transferred from en.wikipedia to Commons by Vojtech.dostal.), CC BY 2.5, https://commons.wikimedia.org/w/index.php?curid=17968876, accessed on 7 March 2024).

Transferrin (Tf) is a serum protein that carries iron in the form of ferric ions (Fe^3+^). Tf has two binding sites for Fe^3+^; a non-bound Tf is called apo-Tf, while Tf bound to one Fe^3+^ is called mono-ferric, and two Fe^3+^ molecules di-ferric or holo-Tf [169]. Tf-Fe^3+^ complexes are delivered to cells via transferrin receptor 1 (TfR1), expressed on the cell surface of most cells in the body [170]. A second TfR has also been identified, TfR2, which has a lower affinity for Tf and is not as widely expressed, mainly in the liver [171]. TfR1 is a transmembrane glycoprotein composed of two identical subunits of 90 kDa each, linked with disulfide bonds. Each subunit has three extracellular domains: the apical (A), the protease-like (P), and the helical domain (H). The helical domain has the leading sites for holo-Tf [172]. Holo-Tf binds with high affinity to TfR1 at physiological pH 7.4. After binding, the holo-Tf-TfR1 complex is internalized via clathrin-coated endosomes. The endosomes are acidified to pH 5.5, which results in dissociation from TfR1 and delivery of iron to the cell [169] (Figure 2).

### 4.3. Engineered Antibody

Antibodies can be engineered to bind to TfR directly or through transferrin. When engineering the antibody, it should preferably bind to a different epitope than Tf on the TfR to avoid interference with the endogenous process of iron delivery to the brain. Many TfR antibodies bind the apical domain of TfR [173,174]. It should have a moderate affinity for TfR in the nanomolar range [175]. High affinity might result in a lower ability of the antibody to dissociate from TfR, which leads to sorting into lysosomes for degradation [176]. The dissociation constant might be more critical than the association constant [175]. Too low affinity might lead to poor ability of the antibody to bind TfR at the BBB and, consequently, poor brain delivery [177]. Affinity can be pH-dependent, so the antibody binds TfR well at physiological pH but dissociates at lowered pH in the early endosome [178].

The dose of the antibody is also an essential factor. Higher affinity can increase brain uptake at low doses, while for high doses, as used in therapy, a lower affinity for TfR increases brain uptake [178]. At non-saturable low doses, binding and brain uptake depend on affinity. In contrast, at saturable doses, lower affinity could be critical for the dissociation from TfR and entry to the parenchyma.

The valency of the antibody to TfR is crucial. A monovalent interaction with TfR is preferable for high parenchymal delivery [179]. A bivalent binding to TfR is more robust due to high avidity. It could cause clustering of TfR on the capillary cells and lead to intracellular sorting to the lysosomes rather than transcytosis [180]. A bivalent antibody can also decrease TfR levels in cells [174].

### 4.4. Transferrin Conjugation

Conjugating with transferrin offers the best option of all the choices available for RMT. There are several considerations in designing this conjugate. First, the suitable conjugation site should be identified on both the antibody and transferrin to enable access to form a stable linkage without compromising the function of either protein. Commonly targeted sites on antibodies include lysine residues that are abundant on the surface and reactive due to their amine groups. Thiol groups are less plentiful but can be used. There are two ways an antibody or a fragment can be conjugated: by allowing the engineered antibody to bind with transferrin in vivo and the other is by binding the antibody and transferrin protein chemically.

Iron, insoluble as free Fe^3+^ and toxic as free Fe^2+^ is distributed through the body as Fe^3+^ bound to transferrin (Tf) for delivery to cells by endocytosis of its complex with transferrin receptor (TfR). Transferrin has two specific iron-binding sites in the N-lobe (N-terminal lobe) and C-lobe (C-terminal lobe). Each site can reversibly bind one ferric ion (Fe^3+^) along with a carbonate anion and function independently. The N-lobe (transferrin A) is part of the N-terminal lobe of transferrin. It binds iron when transferrin interacts with its receptor on the surface of cells, specifically in areas where the pH is slightly more acidic, facilitating iron release. The C-lobe (transferrin B) is located in the C-terminal lobe. This site also binds iron under similar conditions but functions independently, meaning the iron release and binding can occur at either site without necessarily affecting the other [181]. Table 3 lists the FASTA sequence of transferrin bound to receptor (PDB 1SUV). The C-lobe is the target of binding to the target protein for transcytosis:

### 4.5. In Vivo Linker

The first attempt for in vivo binding is the monovalent anti-TfR conjugate based on Roche’s “brain shuttle” technology applied to gantenerumab (Trontinemab) (RG6102), shows an 8-fold higher CSF-to-serum ratio than standard gantenerumab. This Fab fragment binds to the transferrin receptor and is attached to the gantenerumab monoclonal antibodies effector (Fc) domain. This leads to its endocytosis and release of antibodies into the brain parenchyma. This leads to its endocytosis and release into the brain parenchyma [182]. However, this approach has several drawbacks. First, the binding with transferrin in vivo will always be unpredictable, subject, and highly subjective. Second, the binding nature is specified by the structure of the Fab, which can only be based on natural linkers available in the body. These noncleaving linkers result in exocytosis as the conjugate inside the cell binds to the receptor and thus returns to general circulation. This property reduces the therapeutic efficacy. While it is anticipated that the Fab binding to transferrin still allows iron binding, this cannot be ensured, possibly affecting the iron transport cycle. As discussed below, the linker between the antibody and its fragment must be cleavable, so upon entering the brain, the linker is broken, blocking the antibody’s or the fragment’s exocytosis.

### 4.6. Fc-Domain Engineered Transcytosis

Much progress has been made in developing receptor-mediated transcytosis (RMT). Previous RMT-based delivery strategies have relied upon antibody variable domain binding to engage brain endothelial cell receptors [20,165,183]. Most of these approaches use one of two architectures: a bispecific antibody where one Fab binds the RMT receptor and the other binds a therapeutic target [184] or an antibody where both Fabs bind a therapeutic target and an RMT-binding domain is appended to a heavy or light chain terminus via a peptide linker [185]. These approaches, respectively, preclude bivalent and bispecific therapeutic targeting or require the appendage of an unnatural fragment to the immunoglobulin G (IgG) scaffold.

Another novel approach is to engineer an Fc domain to bind to the TfR, enabling brain delivery of biotherapeutics. It starts with generating various libraries, wherein contiguous positions on the Fc are randomized, and then sorting those libraries to isolate TfR binders [186]. The engineered Fc fragment exploits receptor-mediated transcytosis for CNS delivery by binding a highly expressed brain endothelial cell target to bind the apical domain of the human transferrin receptor (hTfR) without the use of amino acid insertions, deletions, or unnatural appendages. Recombinant expression of the engineered Fc domain fused to anti–β-secretase (BACE1) Fabs yielded molecules with native immunoglobulin G (IgG) structure and stability, resulting in substantially improved CNS uptake and sustained pharmacodynamic responses. This transfer platform can accommodate numerous configurations, including bispecific antibodies and protein fusions, yielding a highly modular CNS delivery platform [186].

In another instance, iduronate 2-sulfatase, a lysosomal enzyme deficient in mucopolysaccharidosis type II (MPS II), fused to a moderate-affinity, monovalent TfR-binding enzyme showed enhanced brain exposure from internalization by parenchymal cells and significant substrate reduction in the CNS of an MPS II [180].

### 4.7. Manufactured Linked Products

The success of conjugation depends on the nature of the linkers used. It is complex to design a protein linker that breaks specifically in the brain to deliver an antibody conjugated [187] under certain conditions or in the presence of specific, prevalent, active enzymes in the brain environment. Linkers sensitive to enzymes or pH levels unique to the brain environment are referred. Many linkers can be cleaved by specific enzymes [188]. Common examples include matrix metalloproteinases (MMPs) or cathepsins, which might be more active in areas of inflammation or tumor growth within the brain. The brain’s microenvironment might exhibit altered pH levels, especially in diseased states such as tumors. Linkers that degrade under acidic conditions could be engineered to release the antibody when they reach these areas.

The pH-sensitive linkers work based on the pH differences between the blood (pH ~7.4) and the intracellular environment within endosomes (~pH 5.5). Such linkers can remain stable in the bloodstream but cleave in the acidic environment of the endosomes within brain endothelial cells, releasing the antibody once inside the brain. One of the most used pH-sensitive linkers in drug conjugates is the hydrazone bond, which is stable at neutral pH but cleaves in acidic conditions (pH ~5.0 to 6.0). Acetal and ketal linkers are also stable at neutral pH but cleave in a tanacidic environment. Theo orthoester linkers are designed to undergo hydrolysis under mildly acidic conditions, making them useful for applications where a slightly earlier release before reaching the most acidic environments (like late endosomes or lysosomes) is beneficial. The β-thiopropionate linkers are designed to be cleaved by esterases, which can be activated or more accessible in different pH environments, adding another layer of specificity to the release mechanism.

Noncleavable linkers can be particularly useful in applications where prolonged therapeutic agent retention is necessary or premature release could lead to toxicity or reduced efficacy [189]. This stability ensures that the therapeutic payload remains conjugated during circulation and only releases the active drug upon internalization into the target cells, typically through cellular processes such as endocytosis [190]. Upon cellular uptake, the entire drug–linker–carrier complex is often routed to lysosomes where low pH and enzymatic activity can degrade the carrier molecule (e.g., an antibody), ultimately releasing the drug within the target cells. This type of linker is commonly used in developing antibody–drug conjugates (ADCs), where the drug must be released within the target cancer cells, reducing the impact on non-target cells and minimizing systemic side effects [191]. A peptide linker (valine-citrulline p-aminobenzylcarbamate) used in antibody–drug conjugates (ADCs) is stable in the bloodstream but is cleaved by cathepsin B in the lysosome. Phe-Lys is another cathepsin B substrate used in various therapeutic conjugates. It provides a good balance of stability and cleavability.

Suppose the linkers are made part of a recombinantly or mRNA-delivered product as a single molecule comprising the antibody and conjugated transferrin. In that case, a linker like the aspartic acid-proline (Asp-Pro) bond is suitable as it is susceptible to acidic environments due to the susceptibility of the peptide bond to acid-catalyzed hydrolysis. Histidine residues have a side chain with a pKa around 6.0, making them sensitive to pH changes near physiological and lysosomal conditions. However, incorporating multiple histidine residues in a row can create a segment in a peptide linker that becomes charged under acidic conditions, potentially destabilizing the linker, and facilitating cleavage. Other examples of these linkers include [192,193] include:

- Val-Cit (VC): The sequence Valine–Citrulline (Val-Cit) is a commonly used dipeptide linker that is cleavable by Cathepsin B. It is often used in antibody–drug conjugates (ADCs) targeting cancer cells [194].
- Phe-Lys (FK): Phenylalanine–Lysine (Phe-Lys) is another dipeptide sequence that can be specifically cleaved by Cathepsin B [195]. This linker is useful in contexts where a slightly different cleavage rate or stability is required compared to Val-Cit.
- Gly-Phe-Leu-Gly (GFLG): This tetrapeptide sequence is one of the most typical Cathepsin B-sensitive linkers. It offers a balance of stability and efficient cleavage under physiological conditions [196].
- Ala-Leu-Ala-Leu (Ala-Leu)2: This repeated dipeptide sequence provides a robust framework sensitive to Cathepsin B cleavage. It is helpful in formulations where extended linker length is needed for optimal drug function [197].

The choice of non-cleavable peptide linkers will include G4S and other similar peptides that stay connected. Still, these would not be desirable except for connecting antibody fragments’ VH and VL parts.

### 4.8. Nanoparticle

Another approach to transferrin-driven cytosis involves coating nanoparticles such as those in an mRNA composition with transferrin protein [198]. Transferrin can be conjugated to the surface of LNPs through covalent linkage to PEG–lipids or via biotin–streptavidin interactions, where transferrin is biotinylated, and streptavidin is incorporated into the LNP formulation. The density of transferrin on the surface of LNPs needs to be optimized to balance targeting efficiency with the potential for receptor saturation or downregulation.

These LNPs can specifically bind to TfRs, enhancing their uptake through receptor-mediated endocytosis. Following binding, the transferrin-coated LNPs are internalized and can be transported across the endothelial cells via transcytosis; once on the other side of the BBB, the LNPs can release their mRNA cargo within the brain environment, allowing for localized expression of therapeutic proteins.

## 5. An Example of Computer Modeling

To establish the proof of efficacy, pharmacokinetic profiling on a radioactive drug [199] in animal species to demonstrate preferred accumulation in the brain is a well-defined technique; the process involves making the current product radioactive and conjugating it with transferrin in vitro and then comparing the radio image with the same molecule but without conjugation. Importantly, these studies can be executed swiftly and with minimal costs without requiring regulatory oversight.

**Figure 3:**
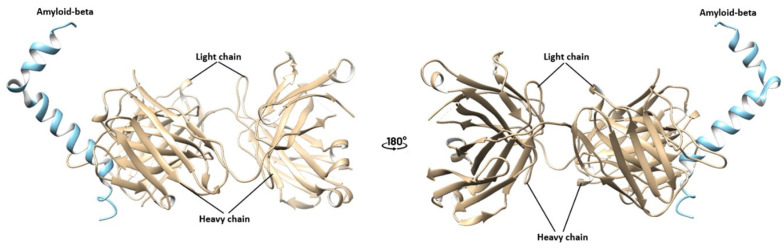

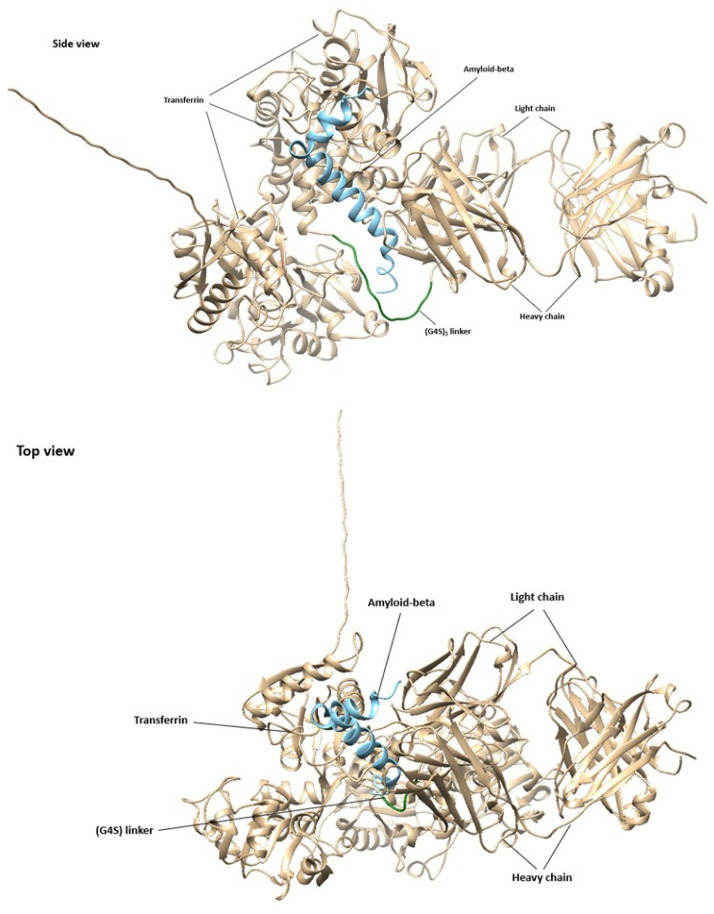
Amyloid-beta (blue) bound to aducanumab (tan) (**top**); and with transferrin: **middle**, bound with long linker and **bottom** bound with short linker.

Before conducting animal studies, it is imperative to check that binding with transferrin does not hinder the binding of the antibody with amyloid-beta. To prove this, we employed bioinformatic modeling to further characterize the drug’s behavior and investigate the impact of non-cleavable linkers, such as (G4S)n, on antibody binding. Our examination, centered on steric hindrance, sought to understand the effects of the length of the linker and its influence on brain penetration. The complex formed between aducanumab and Aβ has already been documented in the Protein Data Bank (PDB ID: 6c03) [200,201]. Leveraging this information, we conducted modeling experiments focusing on aducanumab, an Alzheimer’s disease treatment previously withdrawn from the market. Our methodology involved pre-processing and standardizing the antibody structure using UCSF Chimera, protein structure prediction using the AlphaFold2, followed by docking analysis using HADDOCK, both before and after linkage with transferrin via the (G4S)n linker. Subsequently, we evaluated binding affinity and interaction patterns through the PRODIGY server, mainly the linker’s length (Figure 3).

Our protein–protein complex analysis results reveal significant differences in binding affinity and interaction characteristics (Table 4). The ΔG value was calculated at −18.1 kcal mol^−1^, with a corresponding Kd of 5.10 × 10^−14^ M at °C for aducanumab–Aβ complex. The interface analysis demonstrated varying interactions, with notable contributions from charged–charged and apolar–apolar interactions. We observed enhanced binding affinity and altered interaction profiles upon conjugation with transferrin using different linkers. Specifically, the transferrin-(G4S)_3_–aducanumab–abeta complex exhibited the highest ΔG value (−29.2 kcal mol^−1^) and the lowest Kd (4.10 × 10^−22^ M at °C), indicative of stronger binding. Interface analysis further revealed increased interactions across all categories, particularly in polar–apolar and apolar–apolar interactions. These findings underscore the importance of linker length in modulating protein binding and highlight the potential of our approach for optimizing drug–protein interactions. These findings underscore the critical role of linker length in modulating binding properties. Comparative analysis using (G4S) or (G4S)_3_ linker has revealed superior flexibility with the latter, facilitating unimpeded binding to proteins with minimal steric hindrance around the amyloid-binding pocket. Furthermore, these findings indicate that linking the antibody’s light chain with transferrin does not compromise antibody binding to brain proteins nor impede amyloid’s accessibility to its binding domain.

## 6. Conclusions

NDs frequently involve disordered proteins that the inefficient immune system of the brain is not capable of removing, leading to scores of untreatable disorders. Much of current research is focused on designing antibodies, and a few have been approved, yet their use remains limited due to their poor entry into the brain, even as nanobodies. The effectiveness of these antibodies can be substantially increased if they are conjugated with transferrin protein as a choice modification to enhance their entry into the brain.

Currently, the dose of antibodies entering the brain is less than 0.1%; thus, any change brought by improved transit across the brain will dramatically change their efficacy. In our opinion, this modification should be a standard approach for all future treatments since this provides a more reproducible means of promoting the entry of antibodies into the brain compared to dozens of other invasive and noninvasive techniques [202].

We propose reviving the antibodies that have failed in efficacy trials against the NDs should be rejuvenated by reengineering them to smaller-sized fragments and conjugating with transferrin. Such conjugations do not alter the binding properties of antibodies or their fragments, as demonstrated with the example of aducanumab.

The development plan presented is practical and inexpensive, deserving the full attention of the developers of antibodies against the NDs that have so far been disappointing. The FDA has begun to realize it and is now seeking public opinion about the utility of full antibodies in the NDs treatment; in the case of donanemab, a meeting will be held on 15 June 2024; the findings will reported soon by the FDA [203]. If approved, donanemab, a humanized IgG1 monoclonal antibody, would be the third anti-amyloid therapy to gain FDA approval, following aducanumab (Aduhelm; Biogen) in 2021 and lecanemab (Leqembi; Eisai) in 2023. Aducanumab was pulled from the market. We have filed comments to the FDA to reject the approval of donanemab.

## Figures and Tables

**Table 1:** Approval and structural attributes of anti-Aβ monoclonal antibodies [58,59,60].

Antibody (Structure or Part)	IgG Subclass	Sequence Targeted	Aβ Conformation Specificity	Highest Trial Phase Completed
Bapineuzumab PDB 4OJF Fab complexed to amyloid beta 1–8 [61]	IgG1 (humanized mouse mAb 3D6)	Aβ1–5	Monomers, oligomers, fibrils	III, failed
Solanezumab (PDB 4XXD; Kegg D10058) [62]	IgG1 (humanized mouse mAb m266)	Aβ16–26	Monomers	III, failed
Crenezumab (PDB 5VZX; Kegg D10101) [63]	IgG4 (humanized)	Aβ13–24	Monomers	III, failed
Gantenerumab (PDB 5CSZ; Kegg D09713) [63]	Human IgG1	Aβ2–11 and Aβ18–27	Oligomers, fibrils	III, failed
Aducanumab (PDB Fabs complex to Aβ 8azs; 6CNR; 6CO3; KEGG 10541;	Human IgG1: human-derived monoclonal antibody	Aβ3–7	Oligomers, fibrils	Approved and withdrawn
Lecanemab (PDB 8AZU; KEGG D11678 [60]	IgG1 (humanized mouse mAb158)	Aβ1–16	Oligomers, protofibrils, fibrils	Approved
Donanemab (PDB 7CM0; KEGG D11500) [60]	Humanized IgG1 (developed from mouse IgG2a mE8)	Aβ3–42	Fibrils	III; halted at FDA
Cinpanemab (PDB 6CT7; KEGG D 11467) [60]	Human IgG1 human-derived monoclonal antibody against α-synuclein	α-synuclein residues 1–10; 800× affinity for aggregate	Monomer, aggregates	III halted by the developer
Gantenerumab PDB 5CSZ Fragment in complex with Aβ_1–11_; KEGG D09713 (heavy chain only) [60]	Human IgG1 human-derived monoclonal antibody against Aβ	Aggregated beta-amyloid fibers.	Fibrils	III, failed
Semorinemab, RO7105705 [64,65]	IgG4 monoclonal antibody against Aβ	Anti-Tau	Tau-isoforms	II
Tilavonemab [66]	IgG4 monoclonal antibody against Aβ	Anti-Tau	Tau-isoforms	II
ABBV-8E12 [67]	IgG4 monoclonal antibody against Aβ	Anti-Tau	Tau-isoforms	II
AN179, [68,69]	IgG4 monoclonal antibody against Aβ _42_	Anti-Aβ_4_	Monomer, aggregates	Discontinued after Phase 2

**Table 2:** Molecular weight and half-life of antibodies. Listing in red is below the glomerular filtration rate limits (40 kDa) and thus passes through kidneys readily.

Antibody Type	Molecular Weight (kDa)	Molecular Size (nm)	Disposition Half-Life
Whole IgG	~150	10–15	21 days (average) *
F(ab’)2 fragment	~110	5–10	1 week (approx.)
Minibody (scFv-CH)	~80	4–8	1–3 days (approx.)
rIgG	~75	5–8	1–3 days (approx.)
Fab fragment	~50	3.5–4.5	1–2 days (approx.)
Diabody	~50–60	2–3	Hours to 1 day
Single-chain variable fragment (scFv)	~25–30	2–3	Hours
Nanobody (sdAb)	~12–15	1.5–2.5	Hours to 1 day

* Long half-lives attributed to their recycling by FcRn. Combining smaller antibodies with transferrin increases the molecular weight above the glomerular filtration cutoff point.

**Table 3:** FASA sequence of PDB 1SUV.

1SUV_1|Chains A, B|Transferrin Receptor Protein 1|Homo Sapiens (9606)
LYWDDLKRKLSEKLDSTDFTSTIKLLNENSYVPREAGSQKDENLALYVENEFREFKLSKVWRDQHFVKIQVKDSAQNSVIIVDKNGRLVYLVENPGGYVAYSKAATVTGKLVHANFGTKKDFEDLYTPVNGSIVIVRAGKITFAEKVANAESLNAIGVLIYMDQTKFPIVNAELSFFGHAHLGTGDPYTPGFPSFNHTQFPPSRSSGLPNIPVQTISRAAAEKLFGNMEGDCPSDWKTDSTCRMVTSESKNVKLTVSNVLKEIKILNIFGVIKGFVEPDHYVVVGAQRDAWGPGAAKSGVGTALLLKLAQMFSDMVLKDGFQPSRSIIFASWSAGDFGSVGATEWLEGYLSSLHLKAFTYINLDKAVLGTSNFKVSASPLLYTLIEKTMQNVKHPVTGQFLYQDSNWASKVEKLTLDNAAFPFLAYSGIPAVSFCFCEDTDYPYLGTTMDTYKELIERIPELNKVARAAAEVAGQFVIKLTHDVELNLDYEEYNSQLLSFVRDLNQYRADIKEMGLSLQWLYSARGDFFRATSRLTTDFGNAEKTDRFVMKKLNDRVMRVEYHFLSPYVSPKESPFRHVFWGSGSHTLPALLENLKLRKQNNGAFNETLFRNQLALATWTIQGAANALSGDVWDIDNEF
1SUV_2|Chains C, D|Serotransferrin, N-lobe|Homo sapiens (9606)
DKTVRWCAVSEHEATKCQSFRDHMKSVIPSDGPSVACVKKASYLDCIRAIAANEADAVTLDAGLVYDAYLAPNNLKPVVAEFYGSKEDPQTFYYAVAVVKKDSGFQMNQLRGKKSCHTGLGRSAGWNIPIGLLYCDLPEPRKPLEKAVANFFSGSCAPCADGTDFPQLCQLCPGCGCSTLNQYFGYSGAFKCLKDGAGDVAFVKHSTIFENLANKADRDQYELLCLDNTRKPVDEYKDCHLAQVPSHTVVARSMGGKEDLIWELLNQAQEHFGKDKSKEFQLFSSPHGKDLLFKDSAHGFLKVPPRMDAKMYLGYEYVTAIRNLREGTC
1SUV_3|Chains E, F|Serotransferrin, C-lobe|Homo sapiens (9606)
PDPLQDECKAVKWCALGHHERLKCDEWSVTSGGLIECESAETPEDCIAKIMNGEADAMSLDGGYVYIAGQCGLVPVLAENYESTDCKKAPEEGYLSVAVVKKSNPDINWNNLEGKKSCHTAVDRTAGWNIPMGLLYNRINHCRFDEFFRQGCAPGSQKNSSLCELCVGPSVCAPNNREGYYGYTGAFRCLVEKGDVAFVKSQTVLQNTGGRNSEPWAKDLKEEDFELLCLDGTRKPVSEAHNCHLAKAPNHAVVSRKDKAACVKQKLLDLQVEFGNTVADCSSKFCMFHSKTKDLLFRDDTKCLVDLRGKNTYEKYLGADYIKAVSNLRKCSTSRLLEACTFHKH

**Table 4:** The binding properties of various conjugates.

Protein–Protein Complex	ΔG (kcal mol^−1^)	K_d_ (M) at °C	ICs Charged–Charged	ICs Charged–Polar	ICs Charged–Apolar	ICs Polar–Polar	ICs Polar–Apolar	ICs Apolar–Apolar	NIS Charged	NIS Apolar
Aducanumab–Abeta	−18.1	5.10 × 10^−14^	22	20	64	1	19	29	21.2	40.1
Transferrin–(G4S)–Aducanumab–Abeta	−21.3	2.60 × 10^−16^	39	27	83	1	18	43	28.5	35.5
Transferrin–(G4S)_3_–Aducanumab–Abeta	−29.2	4.10 × 10^−22^	32	33	136	7	38	79	28.9	35.7

## Data Availability

https://github.com/Niazi0/Engineered-Antibodies.
